# *Enterobacter hormaechei* in the intestines of housefly larvae promotes host growth by inhibiting harmful intestinal bacteria

**DOI:** 10.1186/s13071-021-05053-1

**Published:** 2021-12-07

**Authors:** Qian Zhang, Shumin Wang, Xinyu Zhang, Kexin Zhang, Wenjuan Liu, Ruiling Zhang, Zhong Zhang

**Affiliations:** 1 Collaborative Innovation Center for the Origin and Control of Emerging Infectious Diseases, Shandong First Medical University (Shandong Academy of Medical Sciences), No. 619, Changchen Road, Taian, 271016 Shandong China; 2 School of Basic Medical Science, Shandong First Medical University (Shandong Academy of Medical Sciences), Taian, 271016 Shandong China

**Keywords:** Beneficial bacteria, Housefly larvae, *Enterobacter hormaechei*, Gut microbiota, 16S rRNA, Microbial interaction

## Abstract

**Background:**

As a pervasive insect that transmits a variety of pathogens to humans and animals, the housefly has abundant and diverse microbial communities in its intestines. These gut microbes play an important role in the biology of insects and form a symbiotic relationship with the host insect. Alterations in the structure of the gut microbial community would affect larval development. Therefore, it is important to understand the mechanism regulating the influence of specific bacteria on the development of housefly larvae.

**Methods:**

For this study we selected the intestinal symbiotic bacterium *Enterobacter hormaechei*, which is beneficial to the growth and development of housefly larvae, and used it as a probiotic supplement in larval feed. 16S rRNA gene sequencing technology was used to explore the effect of *E. hormaechei* on the intestinal flora of housefly larvae, and plate confrontation experiments were performed to study the interaction between *E. hormaechei* and intestinal microorganisms.

**Results:**

The composition of the gut microflora of the larvae changed after the larvae were fed *E. hormaechei*, with the abundance of *Pseudochrobactrum*, *Enterobacter* and *Vagococcus* increasing and that of *Klebsiella* and *Bacillus* decreasing. Analysis of the structure and interaction of larval intestinal flora revealed that *E. hormaechei* inhibited the growth of harmful bacteria, such as *Pseudomonas aeruginosa*, *Providencia stuartii* and *Providencia vermicola*, and promoted the reproduction of beneficial bacteria.

**Conclusions:**

Our study has explored the influence of specific beneficial bacteria on the intestinal flora of houseflies. The results of this study reveal the important role played by specific beneficial bacteria on the development of housefly larvae and provide insight for the development of sustained biological agents for housefly control through interference of gut microbiota.

**Graphical abstract:**

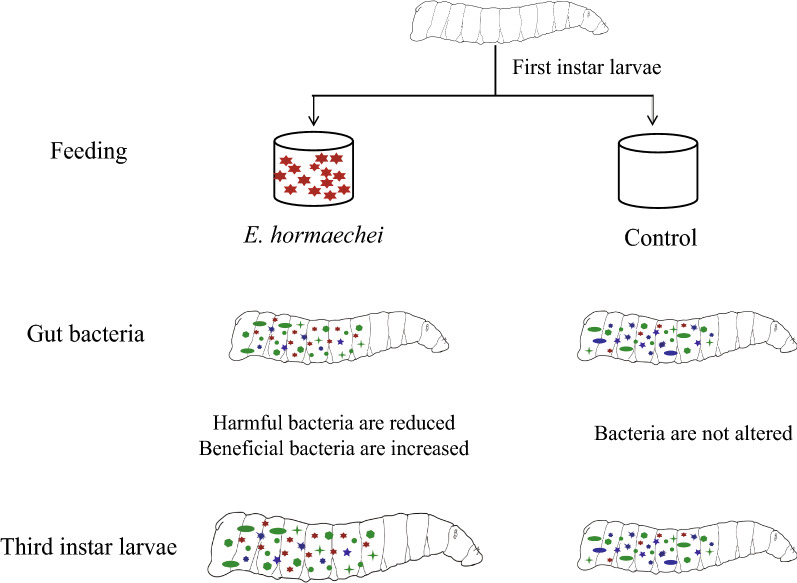

**Supplementary Information:**

The online version contains supplementary material available at 10.1186/s13071-021-05053-1.

## Background

Insects, the largest group of arthropods in the animal kingdom, are one of the most abundant and widely distributed animal groups, inhabiting marine, freshwater and terrestrial habitats ranging from the equator to the poles [1, 2]. Insects are colonized by microorganisms [3, 4], and their guts provide distinctive environments for microbial colonization. To some extent, gut microorganisms contribute to the success of insect diversity and evolution [5]. All microorganisms inhabiting the insect gut are collectively called gut flora, which is the most concentrated interactive group in insects [4]. The gut flora of insects mainly consists of bacteria, most of which are beneficial to the host, protecting the host and defending them against pathogen invasion. These microorganisms are highly dependent on each other, establishing a symbiotic relationship with the host and taking part in the regulation of various life activities of the host. It has therefore been reported that the gut flora could influence the nutrient balance, help digest food ingredients, prevent the invasion of predators, parasites and pathogens and, ultimately, indirectly affect the health of insects [3, 6, 7]. The microbial community the digestive tract of most insects is prominent and plays an important role in the fitness of insects with a variety of lifestyles.

In recent years, in-depth studies of insect symbiotic bacteria have focused on the role of intestinal flora in protecting host insects. Researchers have found that *Serratia marcescens* Y1, an intestinal symbiotic bacterium found in the midgut of *Anopheles sinensis*, renders mosquitoes resistant to *Plasmodium berghei* infection by activating the host mosquito’s immune system [8]. Lignocellulosic herbivorous insects, such as termites and woody cockroaches, can effectively convert lignocellulosic food into sugars, which provide energy for insect growth and development [9]. *Serratia urelytica* Su_Yn1 secretes anti-malaria lipase that selectively kills parasites at various stages, thus providing a new weapon to stop malaria transmission [10]. It has also been reported that feeding probiotics (*Klebsiella oxytoca*) to the Mediterranean fruit fly *Ceratitis capitata* significantly improved the sexual competitiveness of male fruit flies and prolonged their survival [11, 12]. The symbiotic bacteria of *Drosophila melanogaster* (e.g. *Lactobacillus plantarum*) affect mating preference by altering the levels of cuticular hydrocarbon sex pheromones, making this insect more likely to mate with flies with similar gut microbiota [13]. *Pantoea agglomerans*, which has been isolated from locusts, releases large amounts of guaiacol, which inhibits gregarious behavior in locusts [14, 15]. *Blattabacterium*, an endosymbiotic gut bacteria in cockroaches and termites, can utilize nitrogen-containing organic waste for the synthesis of essential amino acids and vitamins and provide nutrients for the host [16]. However, not all intestinal bacteria are beneficial to insects, and some bacteria can enhance pathogen infections. *Serratia marcescens* facilitates mosquito arbovirus infection by secreting a protein named Smenhancin that can digest gut membrane-bound mucins [17]. *Wolbachia* can decrease the developmental time of the Mediterranean fruit fly (*Ceratitis capitata*) larvae and increase mortality [18]. In addition, the gut microbiota can be modulated by increasing the intake of pathogenetic bacteria or reducing the intake of beneficial bacteria, both of which would inhibit the growth of host insect.

The wild housefly (*Musca domestica*) is a global health pest and the vector of many human diseases [19]. However, artificially bred housefly larvae could be utilized as an extremely important resource insect. It can feed on animal waste and biodegrade it to reduce waste disposal [20, 21]. Housefly larvae accelerate the biodegradation of swine manure and improve antibiotic attenuation during vermicomposting [22]. Housefly larval meal as a supplement in livestock fodder and aquaculture feed is considered a potential attractive substitute for protein-rich feed ingredients, and its nutritional value is comparable to that of most high protein feed ingredients [23]. Studies have shown that the dominant gut microflora, including *Providencia*,* Proteus*,* Kurthia*,* Pseudomonas*,* Klebsiella* and *Myroide,*, gradually form during the development of housefly larvae [24]. However, to date, few studies have investigated the effects of gut bacteria associated with the development of housefly larvae. In a previous study we looked at *Pseudomonas aeruginosa* strain Y12 from housefly larvae and demonstrated that low concentrations of *P. aeruginosa* Y12 can protect housefly larvae from *Beauveria bassiana* infections through the production of antifungal compounds [25], while high concentrations of *P. aeruginosa* Y12 significantly inhibit the development of housefly larvae and change the composition and structure of their gut flora. Moreover,* Enterobacter hormaechei* and *Acinetobacter bereziniae* have been found to significantly promote the development of the larvae [26]. However, the effect of feeding beneficial bacteria on the development of housefly larvae has seldom been discussed.

The aim of this study was to investigate the mechanism regulating the influence of specific bacteria on the development of housefly larvae. To this end, we fed *E. hormaechei* to housefly larvae and monitored the changes in their intestinal microbial composition, community structure and interactions with intestinal microorganisms using 16S rRNA gene sequencing technology and plate confrontation experiments. We found that feeding *E. hormaechei* changed the composition and structure of larval gut microflora, increased the diversity of gut microflora and enhanced the stability of gut microflora. Moreover, consumption of *E. hormaechei* inhibited the growth of pathogenic bacteria, such as *P. aeruginosa* and *Providence*, and facilitated the proliferation of beneficial bacteria, thus promoting the growth and development of the housefly larvae. In this study, the influence of beneficial bacteria on the gut flora of the housefly and the relationship between symbiotic gut microorganisms and insects are discussed. Our study highlights the role specific beneficial bacteria would play as microecological agents to improve the utilization efficiency of housefly larvae resources. Additionally, our results revealed that we can interfere with the community structure of the gut flora through inhibiting the growth of some beneficial gut bacteria in the insect pests, thus reducing their survival rate. The results of our research provide insight for the development of novel biological methods to control disease vector pests through the modulation of gut microbiota.

## Methods

### Materials

The houseflies used in this study were from a housefly colony that has been reared in the Laboratory of Vector and Vector-borne Diseases of Shandong First Medical University since 2005. Housefly adults were fed with brown sugar and water, and the larvae were fed with wet wheat bran and milk powder [wheat bran (g):water (ml):milk powder (g) = 1:1:0.4]. The houseflies were raised in an artificial climate incubator maintained at 25 ± 1 °C and 70% relative humidity (RH) under a photoperiod of 12/12 h [light (L)/dark (D)].

*Enterobacter hormaechei* was isolated from the intestines of larvae as follows. Normally reared housefly samples were first soaked in 75% alcohol for 10 min and then cleaned 3 times with sterile double-distilled water to disinfect the body surface. The sample was then ground thoroughly using an automatic grinder and mixed with 100 μl sterile water. A 50-μl sample of the mixture was diluted into three different concentrations (10^–2^,10^–4^,10^–6^), and 100 μl of each dilution was evenly coated onto nutrient agar medium, which was then incubated at a constant 37 °C for 24 h until bacteria colonies were formed. A single colony from each plate was selected based on differences in morphology and other characteristics of bacteria and inoculated onto a new nutrient agar medium; this process of separation and purification was repeated until a single colony was obtained. All experimental operations were conducted under strictly aseptic conditions.

### Experimental design

*Enterobacter hormaechei* was inoculated into freshly prepared Luria–Bertani (LB) medium and incubated in a constant temperature culture oscillator at 37 °C and 110 rpm/min for 24 h, resulting in a concentration of *E. hormaechei* of 6.5 × 10^8^ cfu/ml, which was used in feeding experiment. This feeding experiment consisted of three experiment groups, namely larvae fed sterilized wheat bran supplemented with *E. hormaechei* (Eh), LB medium (Lb) or sterile water (Wa), respectively, in the ratio of 2:1. The LB medium group was used the negative control to provide nutrition for the larvae, and the Wa group was used as control. For the experiment, we used a 10-ml centrifuge tube with a small hole on the top to ensure air permeability. An equal amount of wheat bran supplemented with Eh, Lb or Wa was placed in each centrifuge tube, and then 10 normal-breeding, good-growing, uniformly sized 1-day-old larvae were added to each tube for a total of 150 larvae per group. Each group was set up in three repetitions, and a piece of gauze was placed between the tube and the lid to prevent the larvae from escaping. The tubes were placed in an artificial climate incubator maintained at 25 ± 1 °C, 70 ± 5% RH and a photoperiod of 16/8 h (L/D).

After feeding, three larvae were taken from each tube at the same time each day, and the measurements of biological indexes, such as body length, body weight, pupation rate and emergence rate, were recorded. After removing the surface debris, the larvae were placed in a 1.5-m centrifuge tube containing 75% alcohol, soaked and disinfected for 10–15 min and then rinsed with sterile deionized water 3 times to remove the bacteria attached to the surface of the larvae. This disinfection and rinsing process was repeated 3 times. After strict body surface disinfection, the housefly larvae samples were stored at − 80 °C and sent for high-throughput sequencing. The larvae removed from the different treatment groups and control groups each time were used as a sampling unit, and each sampling unit had three replicates.

In order to further analyze the effects of *E. hormaechei* on larval development and investigate whether the growth differences between Lb and Eh group were due to the nutrition change as a result of *E. hormaechei* proliferation, we conducted experiments in which larvae were treated with sterile water (Wa), Lb-cultured *Providencia stuartii/Providencia vermicola* (Ps/Pv), sterilized Lb-cultured *E. hormaechei* (wEh), co-fed with Lb-cultured *E. hormaechei* and Lb-cultured *P. stuartii/P. vermicola* (Eh + Ps/Pv), sterilized Lb-cultured *E. hormaechei* and *P. stuartii/P. vermicola* (wEh + Ps/Pv), Lb-cultured *E. hormaechei* and sterilized Lb-cultured *P. stuartii/P. vermicola* (Eh + wPs/wPv) and sterilized Lb-cultured *E. hormaechei* and sterilized Lb-cultured *P. stuartii/P. vermicola* (wEh + wPs/wPv). The biological parameters of the housefly larvae were measured using the same methods as described above.

### Interaction between *E. hormaechei* and gut microorganism of housefly larvae

The normally reared housefly samples were soaked in 75% alcohol for 10 min [25, 26, 31] and cleaned with sterile double-distilled water 3 times to disinfect the body surface. The samples were thoroughly ground using an automatic grinder. After gradient dilution (10^–2^,10^–4^,10^–6^), the grinding fluid was inoculated onto nutrient agar medium, placed in constant temperature incubator at 37 °C for 24 h until the bacterial colonies were formed. Based on differences in morphology and other characteristics of bacteria, a single colony from each plate was selected and inoculated into a new nutrient agar medium; this process of separation and purification was repeated until a single colony was obtained. All experimental operations were conducted under strictly aseptic conditions. The cultivable bacteria in the gut of housefly larvae were isolated and purified by this method. To obtain the bacterial culture medium, cultivable gut bacteria were inoculated in LB medium, placed in a constant temperature culture oscillator and then cultured with shaking at 37 °C, 110 rpm/min for 24 h. The *E. hormaechei* culture thus obtained was inoculated on one-half of a nutrient agar plate using the spread plate method with a sterile cotton swab, and the opposite side of the agar plate was used as a negative control. Two 6-mm-diameter sterile filter papers were then symmetrically placed on the two sides of agar medium, and 10 µl of the isolated cultivable gut bacteria, including *Klebsiella pneumoniae*,* Pseudomonas aeruginosa*,* Acinetobacter bereziniae*,* Providencia stuartii*,* Enterobacter cloacae*,* Lactococcus lactis*,* Lysinibacillus fusiformis*,* Providencia vermicola* and *Bacillus safensis*, were added to the filter papers. The plates were cultured at 37 °C for 48 h. The colony sizes of the different isolated bacteria were measured to evaluate the interactions between *E. hormaechei* and the different cultivable gut bacteria. The experiments were conducted with three independent biological replications.

### The influence of the isolated bacteria on the growth of housefly larvae

The gut bacterial culture of housefly larvae (*P. stuartii*,* P. vermicola*) was used as the experimental group, LB medium was used as a negative control group, sterile water was used as the control group; all supplements were mixed with sterilized wheat bran at a ratio of 2:1, respectively. The mixed wheat bran was put into 10-ml centrifuge tubes with small holes on the top for ventilation. Ten 1-day-old larvae were placed in each centrifuge tube, and the tubes incubated in culture chambers maintained at 25 ± 1 °C 70 ± 5% RH and a photoperiod of 12/12 h (L/D). The body length, weight, pupation rate and eclosion rate of the housefly larvae were recorded every day.

### Extraction of the intestine DNA

All samples were sterilized individually with 70% (v/v) and 90% (v/v) ethanol solution for 1 min, respectively, and then rinsed 3 times with sterile water in order to remove bacteria from the larvae surface. The digestive tracts of the insect were extracted and put into a 1.5-ml sterilized centrifuge tube filled with 100 µl double-distilled water and ceramic beads (0.1 mm) for DNA extraction. Intestine samples were homogenized in a tissue lyser (Qiagen, Hilden, Germany) followed by genomic DNA extraction using the Wizard Genomic DNA purification kit (A1120; Promega, Madison, WI, USA). Quantification of total DNA was performed after each DNA extraction using a Nanodrop 2000 spectrophotometer (Thermo Fisher Scientific, Waltham, MA, USA) and 2% agarose gel electrophoresis, respectively. Extracted DNA was stored at − 20 °C until further processing.

### PCR amplification, Illumina MiSeq sequencing and bioinformatics analysis

The hypervariable V3-V4 region of the bacterial 16S rRNA gene was amplified with the primers 341F (5ʹ-CCTAYGGGRBGCASCAG-3ʹ) and 806R (5ʹ-GGACTACNNGGGTATCTAAT-3ʹ) using an improved dual-indexing approach for multiplexed 16S rRNA gene sequencing on the Illumina MiSeq platform; Illumina Inc., San Diego, CA, USA). The PCR mixture (20-μl) consisted of 4 µl 5× FastPfu buffer, 2 µl deoxynucleoside triphosphates (dNTPs) (2.5 mM), 0.8 µl of each primer, 0.4 µl FastPfu polymerase and template DNA (10 ng). PCR cycling was carried out in a GeneAmp 9700 thermocycler (Applied Biosystems, Thermon Fisher Scientific, Waltham, MA, USA) under the following conditions: 95 °C for 5 min; 27 cycles of denaturation at 95 °C for 30 s, annealing at 55 °C for 30 s and elongation at 72 °C for 45 s, followed by an additional elongation at 72 °C for 10 min; and a dissociation stage at the end of the run.

PCR products were detected by 2% agarose gel electrophoresis and purified using the QIAquick gel extraction kit (Qiagen). Library pools were constructed with equal amounts of each PCR product by using the TruSeq Nano DNA LT Sample Prep Kit (Illumina Inc.), which were amplified through the paired-end sequencing on the Illumina MiSeq PE300 platform.

The quality control of the original data was carried out using Trimmomatic v0.39 software (http://www.usadellab.org/cms/index.php?page=trimmomatic). Based on the overlap (minimum: 10 bp) between PE reads after quality control, PE reads were assembled using Flash v1.2.11 software (FLASH: Fast Length Adjustment of SHort reads to improve genome assemblies). Quantitative Insights into Microbial Ecology v1.9.1 software (QIIME; QIIME allows analysis of high-throughput community sequencing data) was adopted for processing, and VSEARCH v2.14.1 software (VSEARCH: a versatile open-source tool for metagenomics) was used for detecting chimera sequences.

Based on a sequence similarity level of 97%, the Uclust method in the QIIME software package was employed to perform operational taxonomic units (OTU) clustering analysis. Based on the Silva reference database (Release138), taxonomic annotations were made for the OTUs of each sample. The Shannon, Simpson, Chao1 and ACE indices of microbial communities were calculated using Mothur (https://mothur.org/). The heatmap was graphed using R software. Common and unique OTUs were intuitively explained by the Venn diagram. Principal coordinates analysis (PCoA) based on the Bray–Curtis dissimilarity and unweighted pair group method with arithmetic mean (UPGMA) tree based on unweighted UniFrac phylogenetic distances were used to determine the difference of beta diversity of bacterial communities in different samples.

Co-occurrence network analysis was based on following the Molecular Ecological Network Analyses Pipeline (MENAP) [27]. OTUs of all samples were retained for analysis, and the number of sequences was log-transformed and analyzed using a random matrix theory-based approach [28]. The edges [i.e. connections between taxa as OTUs] correspond to a significant (positive or negative) correlation between nodes (i.e. taxa as OTUs) [29]. The network was performed using Gephi [30]. Potential keystone driver taxa were identified based on differences in network interactions between the experimental group and control group microbiomes (https://web.rniapps.net/netshift) by using the NetShift method.

### Statistical analysis

The experimental data were analyzed by using Microsoft Excel 2010 (Microsoft Corp., Redmond, WA, USA) and SPSS version 20 statistical software (IBM Corp., Armonk, NY, USA). All data are presented as the mean ± standard deviation. Each treatment consisted of three biological replicates. Pupation rate, emergence rate and developmental duration of housefly larvae among different treatment groups were compared by using one-way analysis of variancefollowed by Fisher’s LSD test, with statistical significance set at *P* < 0.05.

## Results

### Effects of feeding *Enterobacter hormaechei* on the development of housefly larvae

**Fig. 1:**
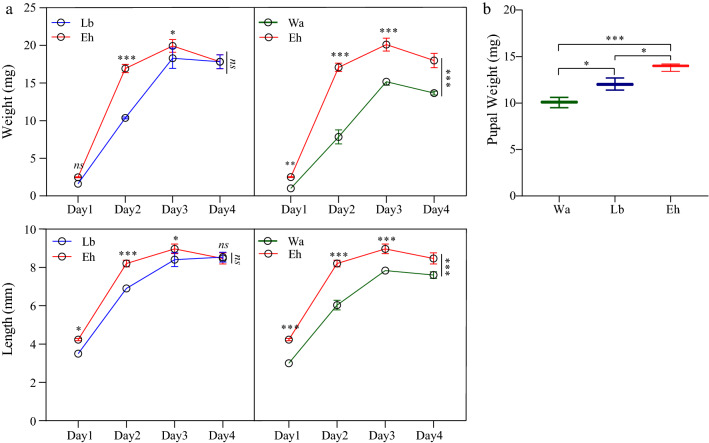
Effects of feeding *Enterobacter hormaechei* to housefly (*Musca domestica*) larvae on their growth and development. **a** Body length and weight of housefly larvae, **b** housefly pupae weight. Repeated measures analysis of variance (ANOVA) followed by Sidak correction was used for multiple comparisons. Asterisks indicate significant difference at *P < 0.05, **P < 0.01, ***P < 0.001. Abbreviations: Eh, *E. hormaechei*; Lb, Luria–Bertani (LB) medium; Wa, sterile water

*Enterobacter hormaechei* (Eh), LB medium (Lb) and sterile water (Wa), respectively, were added to the diet of housefly larvae and the effects of these different diets on the development of the larvae, including their body weight, body length, pupation rate, emergence rate and developmental duration, were analyzed. We found that larvae in the Eh group had a significantly higher body weight (18.85% and 53.13%, respectively) and body length (9.27% and 22.07%, respectively) than larvae in the Lb and Wa groups (Fig. 1a). Also, larvae in the Eh group had increased pupal weight, increased pupation and emergence rates and a shortened growth cycle (Fig. 1b; Table 1). These results indicate that feeding *E. hormaechei* to housefly larvae could significantly promote their growth and development.

**Table 1:** Effects of *Enterobacter hormaechei* on the pupation rate, emergence rate and developmental duration of housefly larvae

Treatment group	Pupation rate (%)	Emergence rate (%)	Developmental duration (d)
Sterile water (Wa)	66.70 ± 4.60 a	84.00 ± 5.40 a	6.50 ± 0.00 a
Luria–Bertani medium (Lb)	76.00 ± 4.00 b	86.00 ± 3.00 a	6.17 ± 0.29 a,b
*E. hormaechei* (Eh)	90.70 ± 5.00 c	91.20 ± 0.40 b	5.83 ± 0.29 b,c

Values followed by different lowercase letters within the same column (developmental parameter) are significantly different, indicating treatment had a significant effect on that parameter

### Analysis of DNA sequences and microbial diversity indices of different housefly larvae samples

**Fig. 2:**
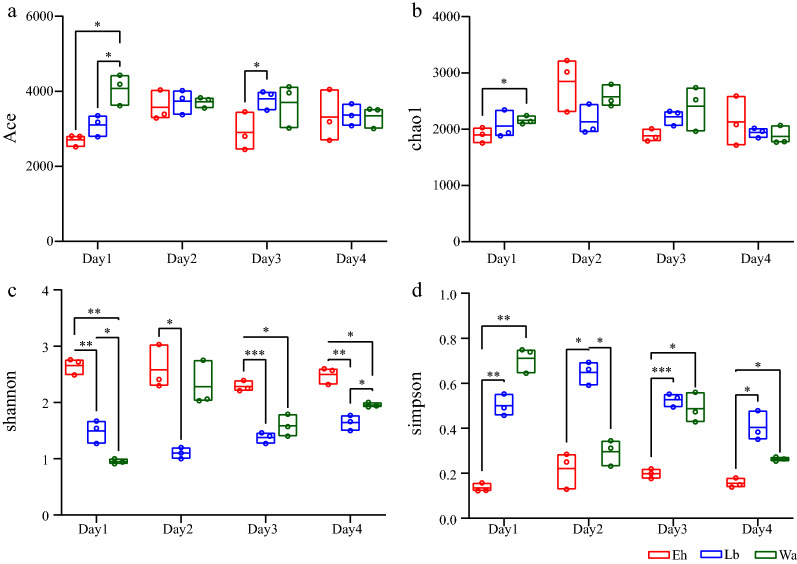
Boxplot of microbial species richness (**a**, **b**) and species diversity (**c**,** d**) indices of different housefly larvae samples. **a** ACE index,** b** Chao1 index,** c** Shannon index,** d** Simpson index index. Data were compared by using two-way ANOVA. Significance analysis was performed using Fisher’s LSD test. Asterisks indicate significant difference at *p < 0.05, **p < 0.01, ***p < 0.001

To further clarify the effect of feeding *E. hormaechei* to housefly larvae on their gut flora, we used 16S rRNA gene sequencing technology to analyze the dynamic changes in gut flora. First, a total of 770,052 high-quality reads were measured from the original data after mass filtration. On the basis of 99% sequence homology, 12,532 OTUs were detected in all samples, of which 3602, 4205 and 4725 OTUs were detected in the Lb, Wa and Eh groups, respectively. The α-diversity indices were used to analyze bacterial community richness and diversity. Analysis of the ACE and Chao1 indices as indicators of species richness showed that the gut bacterial community richness of housefly larvae feeding on *E. hormaechei* were lower than those feeding on LB medium and sterile water after 1 day but that the community richness increased after 2 days in housefly larvae feeding on *E. hormaechei* and showed a slight reversal for larval development. However, analysis of the ACE and Chao1 indices revealed no significant difference among the different treatment groups after 4 days, indicating that larvae feeding on *E. hormaechei* had a similar level of community richness as those of larvae feeding on sterile water (Wa) and LB medium (Lb) after 4 days (Fig. 2a, b). The Shannon and Simpson indices, as indicators of diversity in the OTUs in samples, showed that there was little increase in the microbial community diversity in housefly larvae in the Eh group with larval development. However, bacterial community diversity in housefly larvae in the Eh group was higher than that in the control group across all stages of larval development (Fig. 2c, d).

### Analysis of the composition and structure of gut flora of housefly larvae

At the phylum level, Proteobacteria was the dominant gut flora in all samples. The abundance of Proteobacteria (80.42%) decreased in the Eh group compared with that in the Lb and Wa groups after feeding on *E. hormaechei* for 4 days. The abundance of phyla Bacteroidetes, Firmicutes and Actinobacteria also increased after feeding on *E. hormaechei* (9.30, 5.66 and 4.60%, respectively), among which the abundance of Bacteroidetes increased significantly in the Eh1d group, the abundance of Firmicutes increased in the Eh3d and Eh4d group and the abundance of Actinobacteria increased in the Eh1d, Eh3d and Eh4d groups (see Additional file 1: Figure S1a). At the family level, Enterobacteriaceae was the dominant flora in the Lb and Wa groups (77.77 and 64.55%, respectively). However, the abundance of Enterobacteriaceae (34.62%) decreased significantly in the Eh3d and Eh4d groups, and the abundance of Brucellaceae, Alcaligenaceae and Enterococcaceae increased in the Eh3d and Eh4d groups (33.21, 9.86 and 8.88%, respectively) (Additional file 1: Figure S1b).

**Fig. 3:**
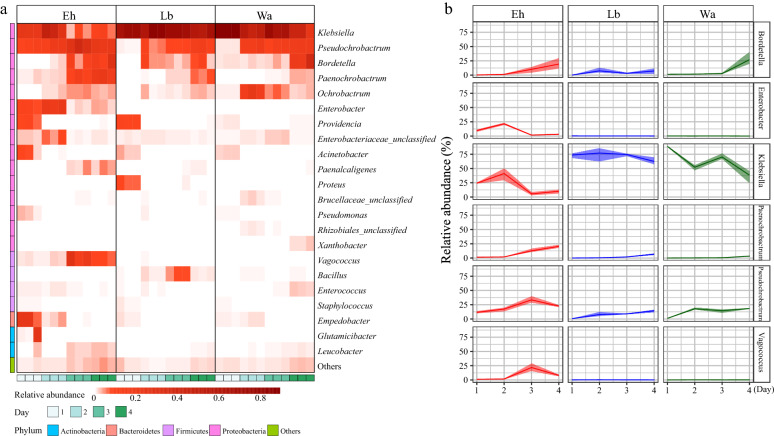
**a** Heat maps of the relative abundances and distributions of bacterial genera in housefly larvae. Heat maps are based on the composition of bacteria genera of the different feeding groups, with each genus color coded, as shown in the panel. **b** Dynamic variation in the relative numbers of key bacteria in the different feeding groups

At the genus level, *Klebsiella* (51.54%) was the most abundant bacterial genera among the 22 dominant genera in the larval samples. The community structure of gut microflora in the Eh group was significantly different from that in the Lb and Wa groups. The relative abundances of *Pseudochromobacter*, *Enterobacter*,* Acinetobacter* and *Empedobacter* in the Eh1d and Eh2d groups significantly increased, while in the Eh3d and Eh4d groups the relative abundances of *Bordetalla*,* Paenochrobactrum*, *Paenalcaligenes*,* Vagococcus* and *Leucobacter* increased. However, the relative abundance of *Klebsiella* and *Bacillus* decreased significantly after feeding on *E. hormaechei* (Fig. 3a), which is consistent with the results of the dynamic analysis of key bacteria (Fig. 3b). The abundance of *Klebsiella*,* Pseudochrobactrum*,* Paenochrobactrum*,* Enterobacter* and *Vagococcus* changed significantly after feeding on* E. hormaechei*. Additionally, we found that the relative abundance of *Paenochrobactrum*,* Bordetella*,* Paenalcaligenes* and *Timonella* increased with the increased larval development, while the relative abundance of *Acinetobacter* and *Empedobacter* decreased with larval development (*P* < 0.05) (Additional file 2: Figure S2).

**Fig. 4:**
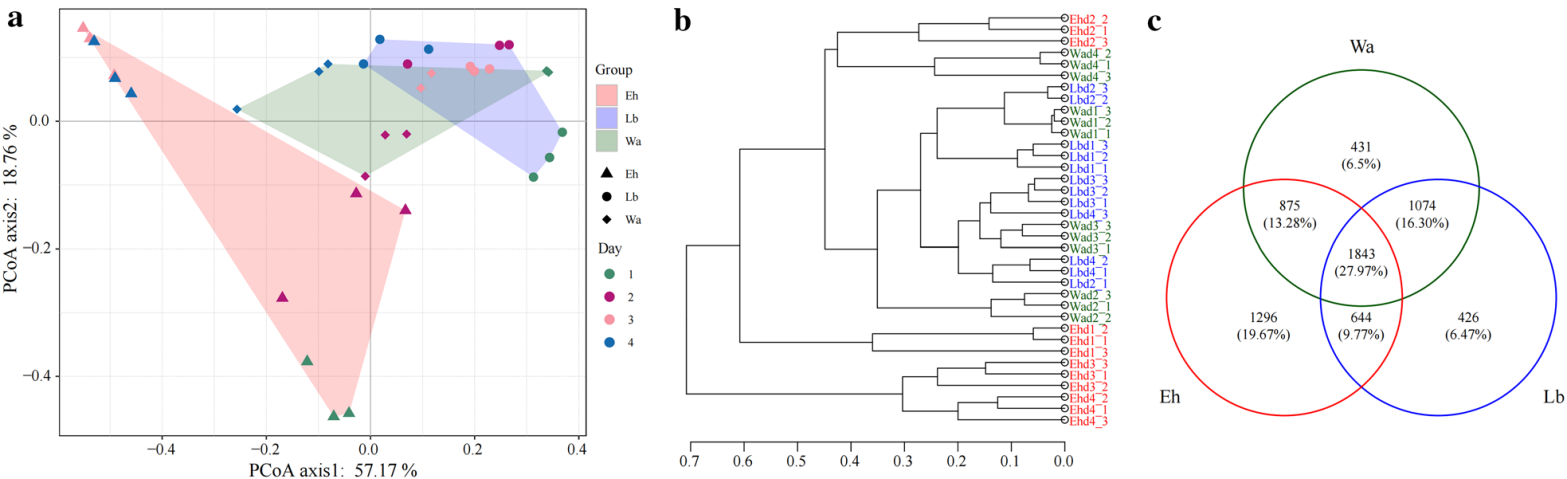
Differences in bacterial community structures and relationships between the feeding groups. **a** Principal coordinate analysis (PCoA) of bacterial community structures of the four groups. Each symbol represents one sample of intestinal bacteria. **b** Unweighted pair group method with arithmetic mean (UPGMA) evolutionary tree analysis of samples. **c** Venn diagram analysis of unique and shared OTUs of the intestinal bacteria in housefly larval samples. The number represents the number of unique OTUs in each sample and common OTUs shared by two or more samples

We further analyzed the structural differences of the gut microflora in the different samples. PCoA showed that the gut microflora structure of housefly larvae in the Eh group was significantly different from that in the Lb and Wa groups, with the gut microflora of the Eh group clustering together, and the gut flora in the Lb and Wa groups clustering together (Fig. 4a). UPGMA tree analysis provided further proof supporting the clustering of samples fed *E. hormaechei* (Fig. 4b). These results demonstrated that *E. hormaechei* significantly altered the gut flora of housefly larvae. The Venn diagram revealed the common and unique OTUs in all samples, among which 1843 OTUs (27.97%) were shared by all samples. There were 1296 (19.67%), 426 (6.47%) and 431 (6.5%) unique OTUs in the Eh, Lb and Wa samples, respectively (Fig. 4c). The Venn diagrams also showed the differences in gut microflora in different samples of housefly larvae.

### Analysis of the interactions of the intestinal flora of housefly larvae

**Fig. 5:**
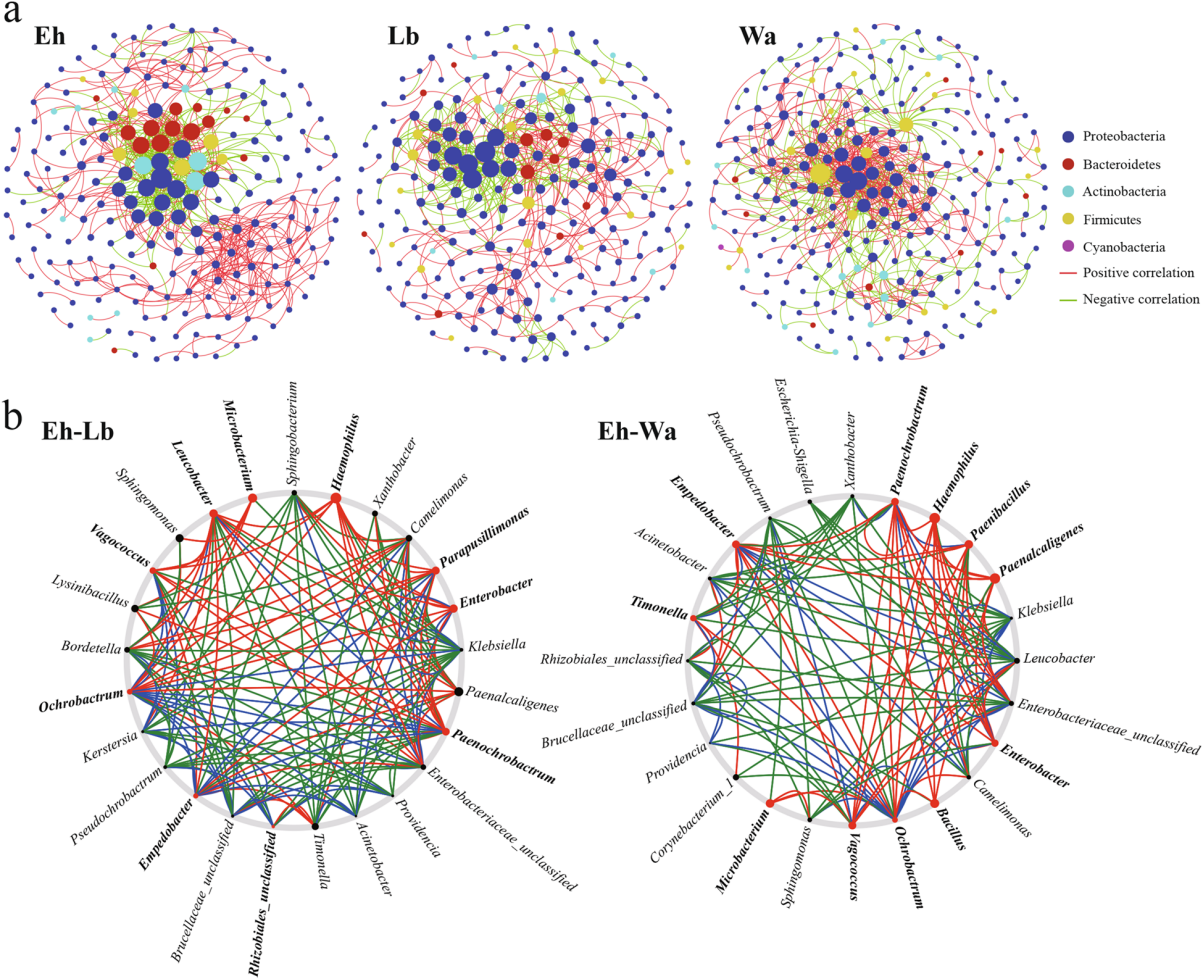
Networks (**a**) and co-occurrence networks (**b**) based on intra- and intergroup intestinal microbiomes. **a** Network analysis between the control groups (Lb, Wa) and the experimental group (Eh). Each point in the figure represents a species, and those species with correlations are connected by a line. The red line represents a positive correlation, the green line represents a negative correlation and the depth of the line represents the level of correlation. **b** Potential “driver taxa” of infection based on bacterial network analysis of the experimental group (Eh) and the control groups (Lb, Wa), marked as Eh–Lb and Eh–Wa, respectively. Node sizes are proportional to their scaled NESH (neighbour shift) score (a score identifying important microbial taxa of microbial association networks), and those nodes colored red were important driver taxa. As a result, large red nodes denote particularly important driver taxa under conditions of *E. hormaechei* feeding. Line colors indicate node (taxa) connections as follows: red edges, association present only in experimental groups; green edges, association present only in control groups; blue edges, association present in both the experimental and control groups

To study the effect of *E. hormaechei* on the interactions of gut microflora of housefly larvae, we first constructed a related network of housefly larvae gut microflora. The results showed that *E. hormaechei* significantly altered the interactions between the intestinal flora of housefly larvae. Compared with the Lb group and Wa experimental groups, the total number of nodes of the interaction network in the gut microflora of the larvae in the Eh group decreased, the average path distance shortened and the average degree and average clustering coefficient increased. Moreover, the positive correlation strength increased and the negative correlation strength decreased in the Eh group, which made the interactions of gut microflora of housefly larvae more stable (Table 2). In all samples, there was a high degree of connectivity within Proteobacteria, especially the interactions between Proteobacteria (85.4%), which were enhanced in the Eh group; the interactions between Bacteroidetes (6.64%), Actinobacteria (4.87%) and Proteobacteria were strengthened, while the interactions between Firmicutes (3.1%) and Proteobacteria, Bacteroidetes and Actinobacteria were clearly weakened (Fig. 5a). The Netshift analysis revealed that *Enterobacter*,* Paenochrobactum*,* Empedobacter*,* Vagococcus*,* Ochrobactrum* and *Haemophilus* were the potential key bacterial groups in the initial microbiomes of housefly larvae fed *E. hormaechei* (Fig. 5b).

**Table 2:** The co-occurrence network indices of the different treatment groups

Treatment group	Network indices							
	Total nodes	Total links	*R*^2^ of power-law	Average degree	Average clustering coefficient	Average path distance	Positive correlation	Negative correlation
*E. hormaechei* ( Eh)	226	707	0.723	6.257	0.205	4.061	65.00%	35.00%
LB medium (Lb)	236	577	0.921	4.89	0.145	4.684	45.23%	54.77%
Sterile water (Wa)	268	715	0.898	5.336	0.171	4.324	64.48%	35.52%

Each treatment included three biological replicates

**Fig. 6:**
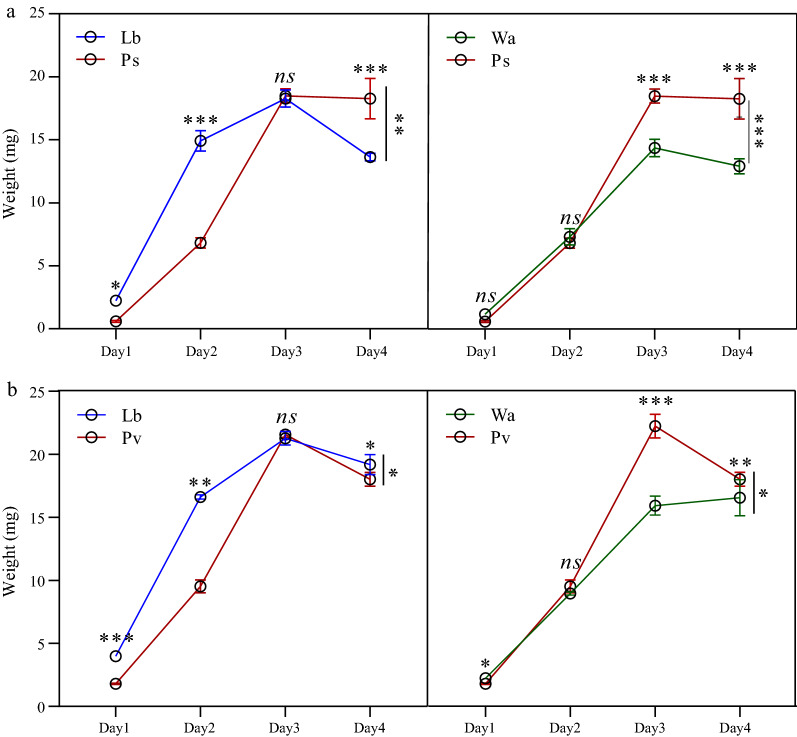
Effects of other cultivable bacteria in the housefly larval intestine on larval development. Repeated measures ANOVA followed by Sidak correction was used for multiple comparisons. Asterisks indicate significant difference at *P < 0.05, **P < 0.01, ***P < 0.001

To further explore the interaction between *E. hormaechei* and cultivable bacteria in the gut of the larvae, we performed plate confrontation assays between different cultivable bacteria (Additional file 3: Fig. S3a). We found that *E. hormaechei* could significantly inhibit the growth of cultivable bacteria such as *P. aeruginosa*,* P. stuartii* and *P. verticola,* in the gut; it also had an inhibitory effect on *K. pneumoniae* and *A. bereziniae* (Table 3). We also carried out a negative verification by the plate confrontation method and a feeding experiment. According to the results of the plate confrontation method, *P. aeruginosa* inhibited the growth of *E. hormaechei*, but *P. stuartii* and *P. vermicola* did not (Additional file 3: Fig. S3b; Additional file 4: Table S1). Previous studies have demonstrated that *P. aeruginosa* can inhibit the growth and development of larvae [31]. Compared with the control group, *P. stuartii* and *P. vermicola* had obvious inhibitory effects on the growth and development of housefly larvae after feeding (Fig. 6a, b).

**Table 3:** Bacteriostatic effects of *E. hormaechei* and cultivable bacteria on the housefly larval intestine

Cultivable bacteria	Control group (mm)	Experimental group (mm)	*t * value	*P * value
*Klebsiella pneumoniae*	10.00 ± 0.00	8.33 ± 0.58	4.00	0.010*
*Pseudomonas aeruginosa*	24.33 ± 1.15	14.00 ± 1.00	11.72	0.000***
*Acinetobacter bereziniae*	9.33 ± 0.58	6.33 ± 0.58	6.36	0.003*
*Providencia stuartii*	10.33 ± 0.58	6.00 ± 0.00	13.00	0.000***
*Enterobacter cloacae*	10.33 ± 0.58	8.67 ± 0.58	3.54	0.020*
*Lactococcus lactis*	7.00 ± 1.00	6.00 ± 0.00	1.73	0.160
*Lysinibacillus fusiformis*	11.00 ± 0.00	9.67 ± 1.15	2.00	0.120
*Providencia vermicola*	10.33 ± 0.58	6.33 ± 0.58	8.49	0.001**
*Bacillus safensis*	10.00 ± 1.00	9.00 ± 0.00	1.73	0.160

Values for the control group and experimental groups are the mean ± standard error of the mean

*P < 0.05, **P < 0.01, ***P < 0.001 (according to Student’s t-test)

**Fig. 7:**
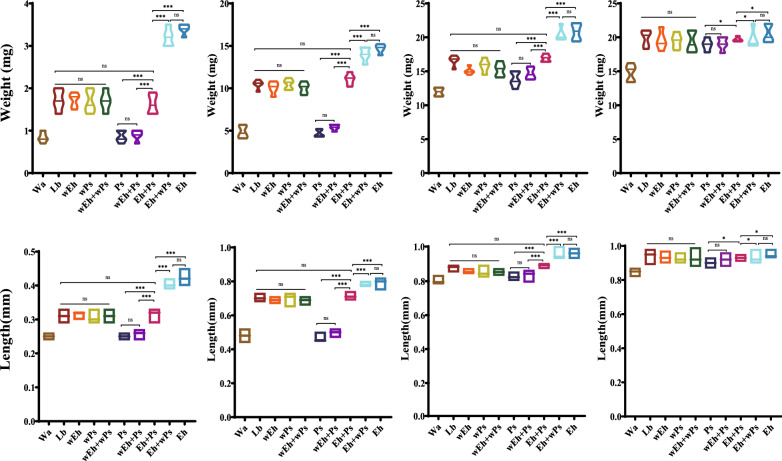
Effects of the *E. hormaechei* and *P. stuartii* on the growth and development of housefly larvae. Housefly larvae were fed with sterile water (Wa), Lb-cultured *P. stuartii *(Ps), sterilized Lb-cultured *E. hormaechei* (wEh), co-fed with Lb-cultured *E. hormaechei *and Lb-cultured *P. stuartii *(Eh + Ps), sterilized Lb-cultured *E. hormaechei *and *P. stuartii* (wEh + Ps), Lb-cultured *E. hormaechei* and sterilized Lb-cultured *P. stuartii* (Eh + wPs) and sterilized Lb-cultured *E. hormaechei* and sterilized Lb-cultured *P. stuartii* (wEh + wPs). Repeated measures ANOVA followed by Sidak correction was used for multiple comparisons. Asterisks indicate significant differences at *P < 0.05, **P < 0.01, ***P < 0.001

In order to further analyze the effects of *E. hormaechei* on larval development, housefly larvae were fed sterile water, Lb-cultured *P. stuartii*/*P. vermicola*, sterilized Lb-cultured *E. hormaechei*, co-fed with Lb-cultured *E. hormaechei* and Lb-cultured *P. stuartii*/*P. vermicola* (Eh + Ps/Pv), sterilized Lb-cultured *E. hormaechei* and *P. stuartii*/*P. vermicola* (wEh + Ps/Pv), Lb-cultured *E. hormaechei* and sterilized Lb-cultured *P. stuartii*/*P. vermicola* (Eh + wPs/wPv) and sterilized Lb-cultured *E. hormaechei* and sterilized Lb-cultured *P. stuartii*/*P. vermicola* (wEh + wPs/wPv). Our results revealed that compared to the control groupof larvae fed sterile water (Wa), the administration of bacterial cultures promoted larval development, indicating that nutrients contained in LB culture medium have positive effects on larval development. However, compared to the sterilized Lb-cultured *P. stuartii* (wPs), Lb-cultured *P. stuartii* (Ps) without sterilization did not show any promoting effects on larval growth after 1–2 days of feeding. In contrast to Lb-cultured* P. stuartii*, Lb-cultured *E. hormaechei* significantly promoted the development and growth of housefly larvae. Moreover, our results revealed that supplementation with Lb-cultured *E. hormaechei* could significantly promote larval development (Eh + Ps) after 1–2 days of feeding compared to the group of larvae only fed Lb-cultured *P. stuartii* (Ps) (Fig. 7). Moreover, based on our our results,* P. vermicola* showed similar effects on housefly development as* P. stuartii* (Additional file 5: Fig. S4).

## Discussion

**Fig. 8:**
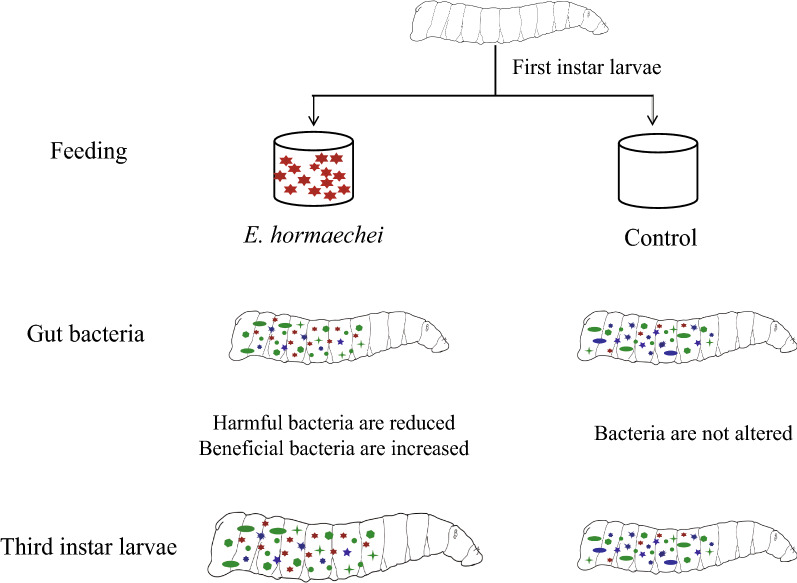
Patterns of growth promotion of housefly larvae by *E. hormaechei*. Different colors represent different bacteria. Red, blue, and green represent *Enterobacter hormaechei*, harmful bacteria and beneficial bacteria, respectively

Symbiotic interactions between insects and microorganisms may have a profound impact on host physiology. [32]. Gut microorganisms that inhabit the intestinal tract of the host larvae play an important role during host development. The addition of beneficial bacteria to the diet provides an ideal nutritional source for larvae and is a promising feeding method [33]. In this study, housefly larvae fed the diet based on *E. hormaechei* showed increased body length, body weight and pupal weight compared to those of the control groups, leading us to assume that the benefits generated in the larval stage may have cascading effects on the fitness, performance and development of both pupae and adults. In addition, the growth cycle of larvae was shortened after feeding on *E. hormaechei*, and this reduction in the length of the developmental cycle is a considerable advantage that can contribute to cost savings and boosted production in large-scale feeding facilities. Previous studies have confirmed that adding *Enterobacter* sp. to the diet can improve the productivity of pupae and adults of *Ceratitis capitata* and shorten the feeding time of males [34]. *Enterobacter* sp. AA26 is considered to be an important component of the insect diet and a potential substitute for beer yeast [35]. Feeding housefly larvae a diet supplemented with *E. hormaechei* did not change the richness of the intestinal flora of the larvae but did significantly increase its diversity, contributing to the health of the houseflies. Previous studies revealed that the taxonomic differences observed in artificially fed flies resulted in reduced adaptability and enhanced sensitivity to environmental changes because of reduced bacterial diversity and functional diversity [36]. Therefore, we speculate that feeding larvae on*E. hormaechei* increased the stability of the gut flora and its adaptability to environmental changes by changing the microbial diversity. This study also further explored the role of Enterobacteriaceae as probiotics. In addition, we analyzed the interactions between *E. hormaechei* and the intestinal microflora of housefly larvae and studied the effect of *E. hormaechei* on the composition and structure of the microbial community of housefly larvae. Our results showed that feeding *E. hormaechei* enriched the composition of gut microflora, promoted the reproduction of beneficial bacteria, inhibited the growth of harmful bacteria and, thus, promoted the development of housefly larvae (Fig. 8).

The bacteria detected in this study belong to four different phylum, among which Proteobacteria was the most abundant. Proteobacteria are found in both larvae and adults of *Bactrocera dorsalis*, which may support their importance in sugar metabolism [37]. The abundance of Bacteroidetes, Firmicutes and Actinobacteria increased after *E. hormaechei* feeding. Actinobacteria is often found in soil-dwelling insects, providing nutrients for insects and protecting them from pathogens as defensive exosymbionts [38]. At the family level, Enterobacteriaceae decreased after *E. hormaechei* feeding, but it was still the dominant flora; Brucellaceae increased significantly. Enterobacteriaceae in insects may indirectly promote host health by preventing the establishment or proliferation of pathogenic bacteria [5, 39]. Enterobacteriaceae (*Citrobacter* sp.) in the intestine of *Bactrocera dorsalis* functions in degrading trichlorphon to improve the resistance of fruit flies to chemical insecticides [40]. Worldwide, Brucellaceae (e.g.* Brucella*) causes brucellosis, which in turn causes Malta fever in humans and abortions in animals [41]. However, the increase in Brucellaceae in this study did not cause the death of housefly larvae, indicating that not all intestinal pathogenic bacteria cause infections in the host, and we speculate that the interactions of microorganisms in the larval gut is one reason for the decline in pathogenic Brucellosis. At the genus level, *Pseudochrobactrum* and *Klebsiella* were dominant strains in the intestines of housefly larvae fed different diets. Compared with the control group, the abundance of dominant bacteria changed significantly after *E. hormaechei* feeding, among which the abundance of *Pseudochrobacter, Enterobacter, and Vagococcus* in the intestine increased significantly. Furthermore, the abundance of *Bacillus* decreased. Previous studies have reported that *Pseudochrobactrum* sp. IY-BUK1 produces enhanced keratinase and protein-rich hydrolysates and has the potential to be used in chicken feather biodegradation [42]. Adding probiotics, such as *Enterobacter* sp., to the diet could change the bacterial load of Enterobacteriaceae in the intestinal tract of *Bactrocera cucurbitae*, reduce the abundance of *Pseudomonas* and significantly improve the quality control parameters of the flies [43]. Probiotic bacteria (*Klebsiella pneumonia*, *Enterobacter* spp. and *Klebsiella oxytoca*) have been found to increase the number of Enterobacteriaceae in the intestine of *Ceratitis capitata* and improve the quality control parameters and sexual function of male flies [11, 44, 45]. Studies have shown that* Bacillus* (e.g. *Bacillus anthracis*, *Bacillus cereus* and *Bacillus thuringiensis*) can produce protein toxins that are toxic to insects, nematodes and mammals [46, 47]. Therefore, we assume that the change in intestinal flora would provide nutrition for the development of larvae, protect the larvae from pathogenic bacterial invasions and promote the growth of the host larvae.

The composition and function of insect gut flora are dynamic, and the interactions between different strains play an important role in insect health and disease. Therefore, we studied the structure of the intestinal microflora and analyzed the interactions between microorganisms of housefly larvae after being fed *E. hormaechei.* We found that the structure of the intestinal microflora changed, and the network interactions between different strains in the intestinal microflora were significantly different in the groups fed or not fed *E. hormaechei*. Compared with the control group, feeding *E. hormaechei* significantly interfered with the gut microbial community structure of housefly larvae. The connectivity between Bacteroides, Actinobacteria and Proteobacteria was highest in gut flora of housefly larvae fed *E. hormaechei* and the positive connectivity level also increased in this group, which further proved that feeding *E. hormaechei* to housefly larvae could promote the stability of their intestinal network. To analyze the interaction between *E. hormaechei* and other cultivable bacteria, we carried out a plate antagonism experiment, and the results showed that *E. hormaechei* significantly inhibited the growth of some cultivable bacteria, such as *P. aeruginosa*,* P. stuartii* and *P. vermicola*. The feeding experiments showed that these three bacterial species had significant negative effects on the growth of housefly larvae. In the gut of housefly larvae fed a large amount of *E. hormaechei*, members of these species may compete for nutrients with other strains and inhibit the growth of *Providencia* and *Pseudomonas* in the intestine of the larvae, reduce the reproduction of harmful bacteria and then promote the growth and development of larvae (Fig. 8). Based on our results, we suggest that the factors accounting for the rapid growth of larvae are: (i) large numbers of *E. hormaechei* consume many nutrients to inhibit the growth of intestinal pathogens; (ii) *E. hormaechei* makes larvae more resistant to pathogens by producing substances that inhibit the growth of pathogens; and (iii) *E. hormaechei* would stimulate the host's immune system to protect the larvae from certain pathogens. The relationship between intestinal symbiotic bacterium and the host immune system in housefly will be investigated in our further research.

## Conclusions

Our study found that the composition, structure and network interactions of the intestinal microflora of housefly larvae changed significantly after larvae were fed *E. hormaechei*. We speculate that *E. hormaechei* inhibited the growth of some pathogenic strains, increased the bacterial load of beneficial flora in the intestinal microbial community, balanced the intestinal flora interactions of housefly larvae and accelerated the growth of housefly larvae. However, the benefits provided by probiotics have not always been consistent according to different studies, most likely due to the complex interactions between gut bacteria and their host insects. To confirm the optimal proportions of probiotics in the diet and avoid potentially harmful effects of higher proportions of probiotics, further studies related to the dose-dependent effects of probiotic feeding on flies are needed.

## Supplementary Information

**Additional file 1: Figure S1. **Microbiome analysis of housefly larvae from different samples at different classification levels. (**a**) and (**b**) represent the relative abundances of bacteria at the phylum and family classification levels, respectively. Wa: sterile water; Lb: Luria-Bertani medium; Eh: *Enterobacter hormaechei*. Day1, Day2, Day3 and Day4 represent the development time of housefly larvae.

**Additional file 2: Figure S2. **Linear regression analysis of OTU number of key bacteria in different groups over time.

**Additional file 3: Figure S3.** Antagonistic experiment between cultivable bacteria in the intestines of housefly larvae. (**a**) Antagonism experiment of *Enterobacter hormaechei* and other cultivable bacteria in the housefly larval intestine. (**b**) Antagonism experiment between *P. aeruginosa*, *P. stuartii*, *P. vermicola *and *E. hormaechei*.

**Additional file 4: Table S1. **Bacteriostatic effects of *P. aeruginosa*, *P. stuartii*, *P. vermicola* and *E. hormaechei* on the intestines of housefly larvae.

**Additional file 5: Figure S4.** Effects of the *E. hormaechei* and *P. vermicola* on the growth and development of housefly larvae. The housefly larvae were fed with sterile water (Wa), Lb-cultured *P. vermicola* (Pv), sterilized Lb-cultured *E. hormaechei* (wEh), co-fed with Lb-cultured *E. hormaechei* and Lb-cultured *P. vermicola* (Eh + Pv), sterilized Lb-cultured *E. hormaechei* and *P. vermicola* (wEh + Pv), Lb-cultured *E. hormaechei* and sterilized Lb-cultured *P. vermicola* (Eh + wPv) and sterilized Lb-cultured *E. hormaechei* and sterilized Lb-cultured *P. vermicola* (wEh + wPv). Repeated measures ANOVA followed by Sidak correction was used for multiple comparisons. Asterisks indicate significant differences at *P < 0.05, **P < 0.01, ***P < 0.001

## Data Availability

All data generated or analyzed during this study are included in this published article [and its additional information files].

## Abbreviations

16S rRNA: 16S ribosomal RNA
OUT: Operational taxonomic units
PCoA: Principal coordinates analysis
QIIME: Quantitative Insights into Microbial Ecology
RH: Relative humidity
