# Supplementary material for: Enterobacter hormaechei in the intestines of housefly larvae promotes host growth by inhibiting harmful intestinal bacteria
Source: Parasit Vectors. 2021 Dec 7;14:598. doi: 10.1186/s13071-021-05053-1 (PMC8653583; doi:10.1186/s13071-021-05053-1)
Supplement: Supplementary file 1 — Additional file 1: Figure S1. Microbiome analysis of housefly larvae from different samples at different classification levels. (a) and (b) represent the relative abundances of bacteria at the phylum and family classification levels, respectively. Wa: sterile water; Lb: Luria-Bertani medium; Eh: Enterobacter hormaechei. Day1, Day2, Day3 and Day4 represent the development time of housefly larvae. [file 13071_2021_5053_MOESM1_ESM.pdf]

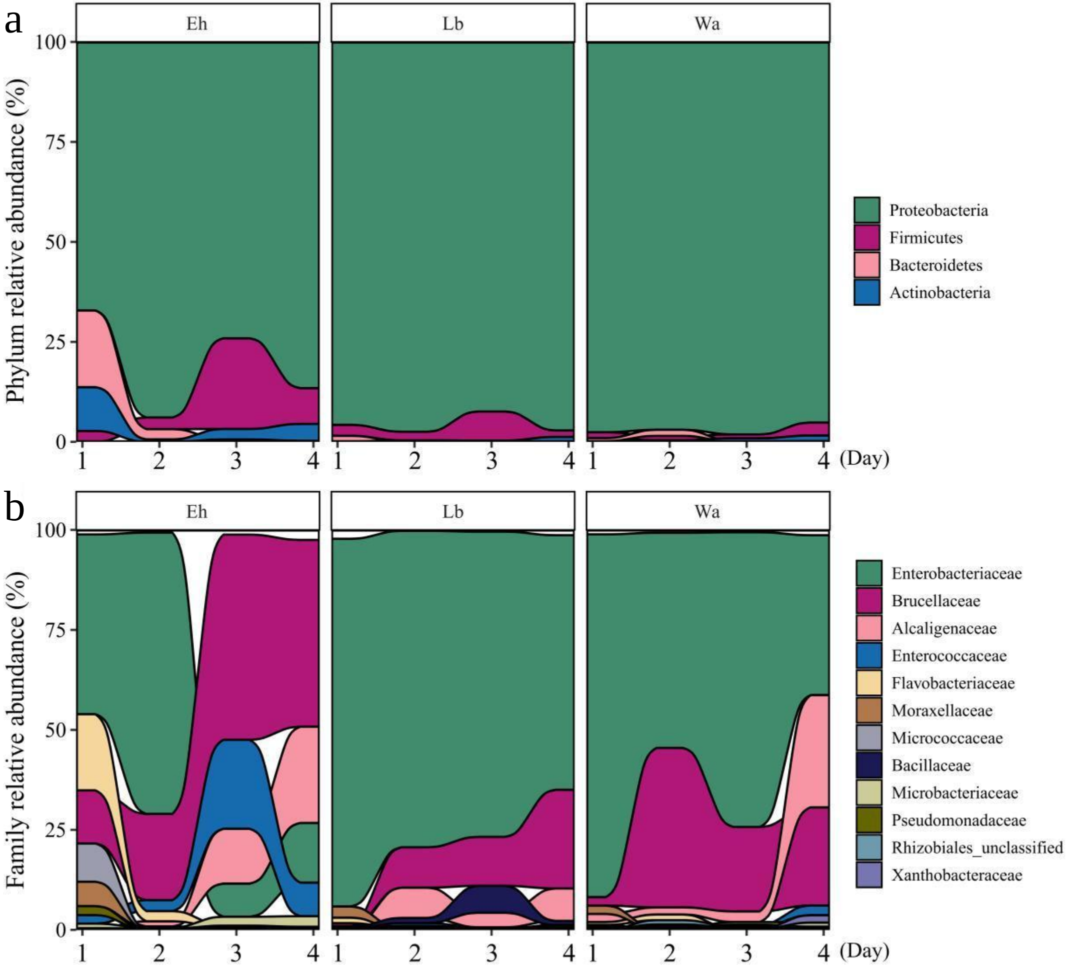

Figure S1. Microbiome analysis of housefly larvae from different samples at different classification levels. (a) and (b) represent the relative abundances of bacteria at the phylum and family classification levels, respectively. Wa: sterile water; Lb: Luria-Bertani medium; Eh: *Enterobacter hormaechei*. Day1, Day2, Day3 and Day4 represent the development time of housefly larvae.
