# Supplementary material for: Enterobacter hormaechei in the intestines of housefly larvae promotes host growth by inhibiting harmful intestinal bacteria
Source: Parasit Vectors. 2021 Dec 7;14:598. doi: 10.1186/s13071-021-05053-1 (PMC8653583; doi:10.1186/s13071-021-05053-1)
Supplement: Supplementary file 2 — Additional file 2: Figure S2. Linear regression analysis of OTU number of key bacteria in different groups over time. [file 13071_2021_5053_MOESM2_ESM.pdf]

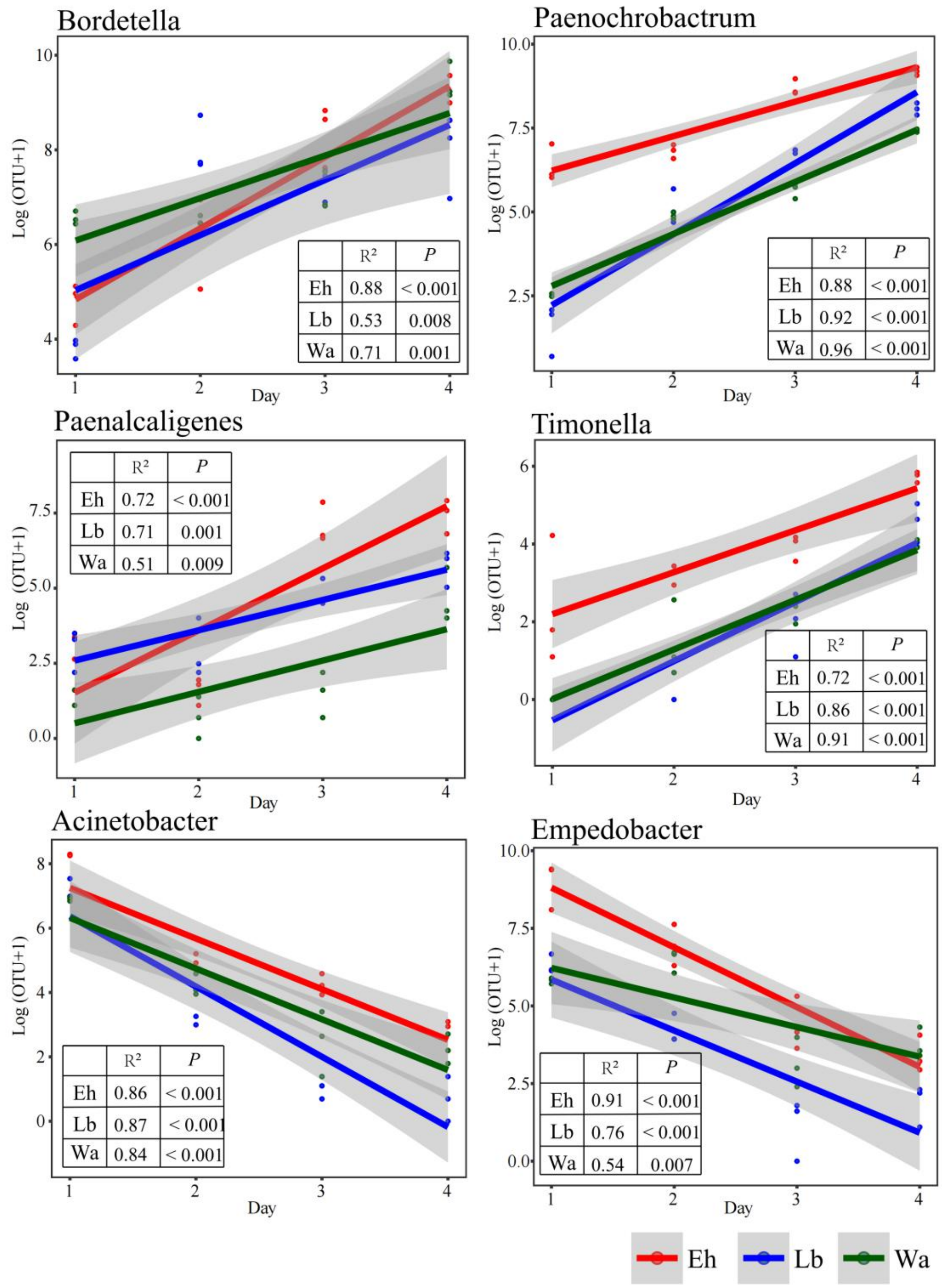

Figure S2. Linear regression analysis of OTU numbers of key bacteria in different groups affected by time. Each treatment included three biological replicates. Wa: sterile water; Lb: Luria-Bertani medium; Eh: *Enterobacter hormaechei*. Day1, Day2, Day3 and Day4 represent the development time of housefly larvae.
