# Supplementary material for: Enterobacter hormaechei in the intestines of housefly larvae promotes host growth by inhibiting harmful intestinal bacteria
Source: Parasit Vectors. 2021 Dec 7;14:598. doi: 10.1186/s13071-021-05053-1 (PMC8653583; doi:10.1186/s13071-021-05053-1)
Supplement: Supplementary file 3 — Additional file 3: Figure S3. Antagonistic experiment between cultivable bacteria in the intestines of housefly larvae. (a) Antagonism experiment of Enterobacter hormaechei and other cultivable bacteria in the housefly larval intestine. (b) Antagonism experiment between P. aeruginosa, P. stuartii, P. vermicola and E. hormaechei. [file 13071_2021_5053_MOESM3_ESM.pdf]

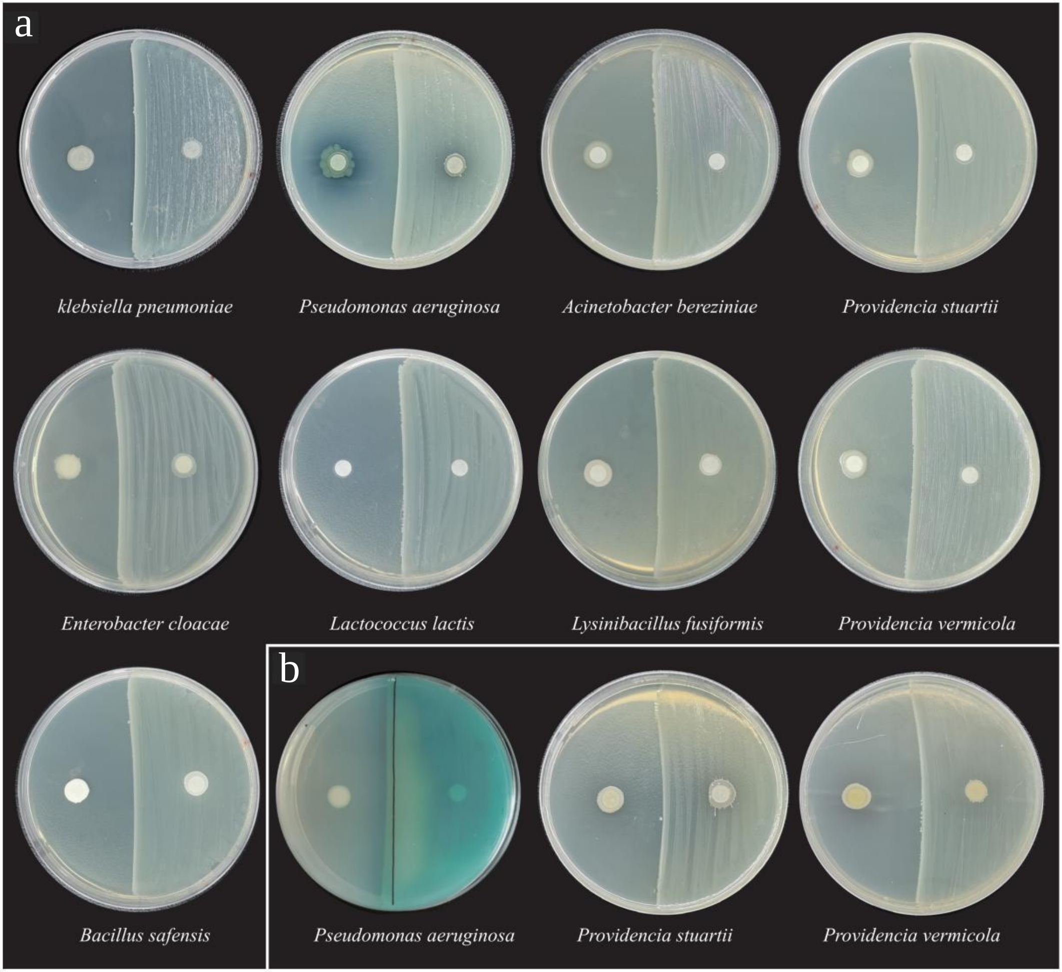

Figure S3. Antagonistic experiment between cultivable bacteria in the intestines of housefly larvae. (a) Antagonism experiment of *Enterobacter hormaechei* and other cultivable bacteria in the housefly larval intestine. (b) Antagonism experiment between *P. aeruginosa, P. stuartii, P. vermicola* and *E. hormaechei.*
