# Supplementary material for: Enterobacter hormaechei in the intestines of housefly larvae promotes host growth by inhibiting harmful intestinal bacteria
Source: Parasit Vectors. 2021 Dec 7;14:598. doi: 10.1186/s13071-021-05053-1 (PMC8653583; doi:10.1186/s13071-021-05053-1)
Supplement: Supplementary file 4 — Additional file 4: Table S1. Bacteriostatic effects of P. aeruginosa, P. stuartii, P. vermicola and E. hormaechei on the intestines of housefly larvae. [file 13071_2021_5053_MOESM4_ESM.pdf]

Cultivable bacteria	Control group/mm	Experimental group/mm	*t* value	*p* value
Pseudomonas aeruginosa	10.33±0.58	6.33±0.58	8.485	0.001**
Providencia stuartii	9.67±0.58	10.00±1.00	0.50	0.6433
Providencia vermicola	9.67±0.58	8.67±0.58	2.12	0.1012

Table S1. Bacteriostatic effects of *P. aeruginosa, P. stuartii, P. vermicola* and *E. hormaechei* on the housefly larval intestines. Each treatment included three biological replicates. The error estimate represents the standard error of the mean. *$p < 0.05$, **$p< 0.01$, ***$p < 0.001$ (Student's t-test).
