# Supplementary material for: Enterobacter hormaechei in the intestines of housefly larvae promotes host growth by inhibiting harmful intestinal bacteria
Source: Parasit Vectors. 2021 Dec 7;14:598. doi: 10.1186/s13071-021-05053-1 (PMC8653583; doi:10.1186/s13071-021-05053-1)
Supplement: Supplementary file 5 — Additional file 5: Figure S4. Effects of the E. hormaechei and P. vermicola on the growth and development of housefly larvae. The housefly larvae were fed with sterile water (Wa), Lb-cultured P. vermicola (Pv), sterilized Lb-cultured E. hormaechei (wEh), co-fed with Lb-cultured E. hormaechei and Lb-cultured P. vermicola (Eh + Pv), sterilized Lb-cultured E. hormaechei and P. vermicola (wEh + Pv), Lb-cultured E. hormaechei and sterilized Lb-cultured P. vermicola (Eh + wPv) and sterilized Lb-cultured E. hormaechei and sterilized Lb-cultured P. vermicola (wEh + wPv). Repeated measures ANOVA followed by Sidak correction was used for multiple comparisons. Asterisks indicate significant differences at *P < 0.05, **P < 0.01, ***P < 0.001 [file 13071_2021_5053_MOESM5_ESM.pdf]

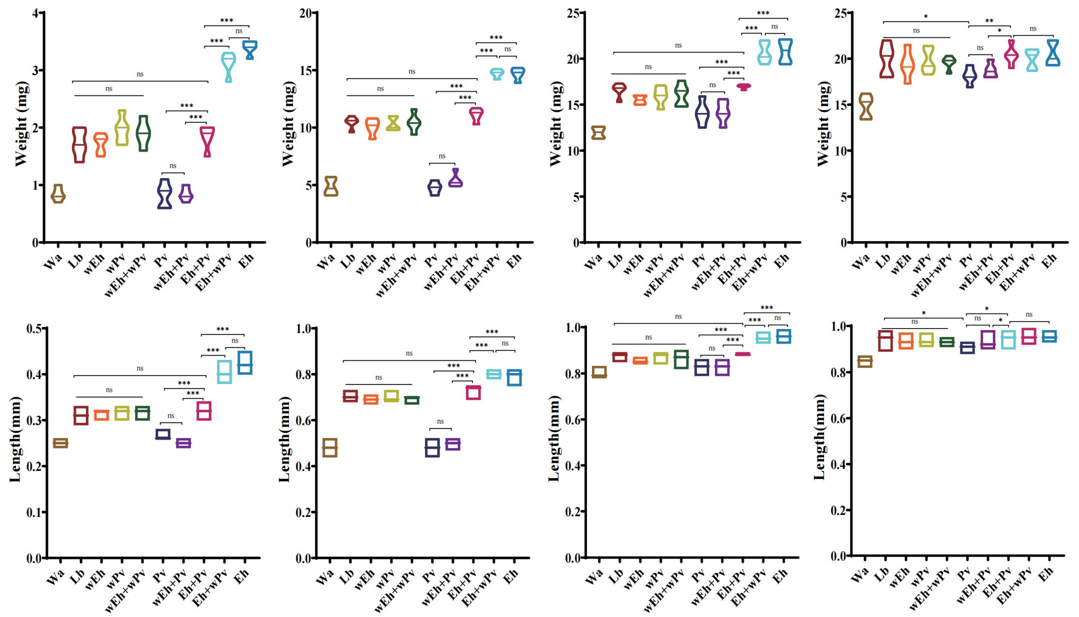

Figure S4. **Effects of the *E. hormaechei* and *P. vermicola* on the growth and development of housefly larvae**. The housefly larvae were fed with sterile water (Wa), Lb-cultured *P. vermicola* (Pv), sterilized Lb-cultured *E. hormaechei* (wEh), co-fed with Lb-cultured *E. hormaechei* and Lb-cultured *P. vermicola* (Eh+ Pv), sterilized Lb-cultured *E. hormaechei* and *P. vermicola* (wEh+ Pv), Lb-cultured *E. hormaechei* and sterilized Lb-cultured *P. vermicola* (Eh+wPv), and sterilized Lb-cultured *E. hormaechei* and sterilized Lb-cultured *P. vermicola* (wEh+ wPv). Repeated measures ANOVA followed by Sidak correction was used for multiple comparisons. **p* < 0.05, ***p* < 0.01, ****p* < 0.001.
